# Supplementary material for: Baseline Dysregulation in B, T, and NK Cells in COVID-19 Predicts Increased Late Mortality but Not Long-COVID Symptoms: Results from a Single-Center Observational Study
Source: Viruses. 2025 Oct 21;17(10):1400. doi: 10.3390/v17101400 (PMC12567989; doi:10.3390/v17101400)
Supplement: Supplementary file 1 [file viruses-17-01400-s001.zip › viruses-3820558-supplementary.pdf]

Supplementary materials

Baseline dysregulation in B, T, and NK cells in COVID-19 predicts increased late mortality but not long-COVID symptoms: results from a single-center observational study

Name and surname

____

PESEL number

____

How long did it take you to recover from COVID-19?

- □ up to 3 months
- □ between 3 and 6 months
- □ more than 6 months

Are your symptoms still present?

- □ yes
- □ no

If your symptoms persist, what are they?

____

Did you experience persistent headaches after COVID-19?

- □ yes
- □ no

Are you currently experiencing persistent headaches?

- □ yes
- □ no

Thromboembolic episode – did deep vein thrombosis occur?

- □ yes
- □ no

Thromboembolic episode – did pulmonary embolism occur?

- □ yes
- □ no

Thromboembolic episode – did a stroke or TIA (transient ischemic attack) occur?

- □ yes
- □ no

Thromboembolic episode – did peripheral arterial thrombosis occur?

- □ yes
- □ no

Have you been diagnosed with an inflammatory joint disease (RA, spondyloarthropathy, PsA)?

- □ yes
- □ no

Have you been diagnosed with a systemic connective tissue disease (SLE, SS, systemic sclerosis, PM or DM)?

- □ yes
- □ no

Have you been diagnosed with a neurological disorder (symptoms appeared after COVID-19), e.g. MS, neurodegenerative disease, chronic headaches, insomnia or sleep disorders, depression or other psychiatric disorders, etc.?

- □ yes
- □ no

If neurological disorders occurred, what were they?

____

Have new cardiac problems appeared (ischemic heart disease, heart failure, arrhythmias/palpitations, fainting)?

- □ yes
- □ no

If cardiac disorders occurred, what were they? Did kidney failure problems appear?

____

Did kidney failure problems appear?

- □ yes
- □ no

If kidney disorders occurred, what were they?

____

Have there been additional episodes of COVID-19?

- □ yes
- □ no

If yes, what was the course? Mild, hospitalized (severe, including ICU)

____

Has the patient been vaccinated against SARS-CoV-2?

- □ yes
- □ no

If vaccinated, how many doses?

____
